# Towards a framework for systematic reviews of the prevalence of exposure to environmental and occupational risk factors

**DOI:** 10.1186/s12940-022-00878-4

**Published:** 2022-07-06

**Authors:** Frank Pega, Natalie C. Momen, Lisa Bero, Paul Whaley

**Affiliations:** 1 Department of Environment, Climate Change and Health, World Health Organization, Avenue Appia 20, 1202 Geneva, Switzerland; 2 General Internal Medicine/Public Health/Center for Bioethics and Humanities, University of Colorado—Anschutz Medical Campus, Denver, CO USA; 3 Lancaster Environment Center, Lancaster University, Lancaster, UK

**Keywords:** Systematic reviews, Systematic review methods, Prevalence, Exposures, Environmental health, Occupational health

## Abstract

Exposure prevalence studies (as here defined) record the prevalence of exposure to environmental and occupational risk factors to human health. Applying systematic review methods to the synthesis of these studies would improve the rigour and transparency of normative products produced based on this evidence (e.g., exposure prevalence estimates). However, a dedicated framework, including standard methods and tools, for systematically reviewing exposure prevalence studies has yet to be created. We describe the need for this framework and progress made towards it through a series of such systematic reviews that the World Health Organization and the International Labour Organization conducted for their WHO/ILO Joint Estimates of the Work-related Burden of Disease and Injury (WHO/ILO Joint Estimates).

We explain that existing systematic review frameworks for environmental and occupational health cannot be directly applied for the generation of exposure prevalence estimates because they seek to synthesise different types of evidence (e.g., intervention or exposure effects on health) for different purposes (e.g., identify intervention effectiveness or exposure toxicity or carcinogenicity). Concepts unique to exposure prevalence studies (e.g., “expected heterogeneity”: the real, non-spurious variability in exposure prevalence due to exposure changes over space and/or time) also require new assessment methods. A framework for systematic reviews of prevalence of environmental and occupational exposures requires adaptation of existing methods (e.g., a standard protocol) and development of new tools or approaches (e.g., for assessing risk of bias and certainty of a body of evidence, including exploration of expected heterogeneity).

As part of the series of systematic reviews for the WHO/ILO Joint Estimates, the World Health Organization collaborating with partners has created a preliminary framework for systematic reviews of prevalence studies of exposures to occupational risk factors. This included development of protocol templates, data extraction templates, a risk of bias assessment tool, and an approach for assessing certainty of evidence in these studies. Further attention and efforts are warranted from scientific and policy communities, especially exposure scientists and policy makers, to establish a standard framework for comprehensive and transparent systematic reviews of studies estimating prevalence of exposure to environmental and occupational risk factors, to improve estimates, risk assessments and guidelines.

## Background

Assessment of prevalence of exposure to health risk factors is a necessary first step in Environmental and Occupational Epidemiology. This knowledge is essential to understand the effects of exposures on health outcomes, or the effects of interventions on decreasing exposures and their attributable disease burden. Environmental and occupational exposures are exposures among persons, which occur in the natural or built environment and at the workplace, to biological, chemical, physical, mechanical and psychosocial risk factors that may impact human health. Prevalence studies of exposure investigate what proportion of a population is exposed to a potential disease agent, and to what level (or intensity) the population is exposed. One example of an occupational exposure is exposure to crystalline silica dust in people working in clothing factories, which is an established physical risk factor for lung cancer among workers. Exposure levels may vary between workers, e.g. due to having worked in the factories for longer, or in roles with more direct or intense exposure. As evidence from these studies grows, it is important that it is synthesised and summarized in an accessible and structured format, tailored for use in environmental and occupational epidemiology research, policy and practice.

Systematic reviews are the gold standard for synthesizing evidence in health research. Systematic reviews on prevalence of exposures within occupational health would answer questions such as: “How many workers were exposed to occupational noise in African countries in 2017?” or “What proportion of workers were exposed to long working hours globally in 2016, and at what levels?”. However, such systematic reviews present a new and challenging frontier for evidence synthesis that must be tackled by research and policy communities, especially exposure scientists and policy makers. No dedicated framework for systematic reviews of the prevalence of environmental and occupational exposures to risk factors to human health currently exists, and existing systematic review frameworks for environmental and occupational health (e.g., related to effects of exposures) are not fully applicable.

In this commentary, we explain the importance of the development of systematic review methodology for studies of the prevalence of environmental and occupational exposures (or, in short, exposure prevalence studies). We outline what such a potential systematic review framework would look like, based on experience of conducting a series of systematic reviews of prevalence of occupational exposures by the World Health Organization (WHO) and the International Labour Organization (ILO), with the support of a large number of individual experts (see [9] for details). These systematic reviews produced the evidence base regarding prevalence and level of occupational exposures for the first WHO/ILO Joint Estimates of the Work-related Burden of Disease and Injury (WHO/ILO Joint Estimates; see https://www.who.int/teams/environment-climate-change-and-health/monitoring/who-ilo-joint-estimates/). We present the progress made thus far, including development of novel tools for assessing risk of bias in exposure prevalence studies and certainty of evidence in systematic reviews of the prevalence of exposures, and make recommendations for the next steps in framework development.

## Main text

### Systematic reviews of prevalence studies of environmental and occupational exposures

Estimates of prevalence of exposure should not come from single or selected studies; full and efficient use should be made of all the available evidence to produce evidence-based health estimates, risk assessments and guidelines. Synthesis becomes increasingly crucial as bodies of scientific evidence grow. With the discipline of Exposure Science rapidly developing, there is a continuously expanding body of evidence on the prevalence of a diverse range of environmental and occupational exposures among relevant populations. The implementation of the US National Academy of Sciences’ vision and strategy for further growing Exposure Science, along with similar initiatives in other countries, can be expected to further accelerate the production of scientific evidence regarding exposure [4].

Systematic reviews maximise transparency and minimise bias when identifying, appraising and synthesizing empirical evidence that is relevant to answering a research question. Systematic review is characterised by: a pre-specified protocol, a comprehensive search strategy, predefined study selection criteria, data extraction, risk of bias assessment and certainty of evidence assessment. Synthesizing prevalence data also enables the exploration of heterogeneity in the data – for example, to consider how and why exposures may differ across workplace settings or work tasks. Not only does evidence need to be synthesised, but it should be made available in a user-friendly format.

While numerous systematic reviews focus on the effects of various environmental and occupational risk factors on health outcomes, systematic review of evidence on the prevalence of environmental and occupational exposures is less common [1]. Previous prevalence reviews often do not fulfil systematic review criteria: focusing on broad topics, rather than a specific research question (for example, “What is the prevalence of occupational exposure to silica, asbestos and coal dust?”, which is the focus of an ongoing WHO/ILO Joint Estimates systematic review [9]); not carrying out a systematic literature search; or not focusing on synthesis of evidence of studies assessed for risk of bias.

The WHO/ILO systematic reviews used a methodology adapted from existing systematic review frameworks. This is possible because basic systematic review principles and fundamental methods are quite generalisable; however, the frameworks needed to be adapted to the specific requirements of prevalence questions and the evidence needed to answer them. The adaptations are summarised in Table 1. The two most extensive adaptations and innovations are with regards to risk of bias assessment, and assessment of certainty in the evidence for a prevalence systematic review.

**Table 1:** Steps from existing systematic review frameworks and modifications needed (if relevant) to apply the steps to systematic reviews of prevalence studies of environmental and occupational risk factors to human health

Framework step	Modifications needed?
Specify research question	Yes – *not usually a comparator, no outcome*
Protocol	No – *basic general step of systematic review*
Systematic search	No – *basic general step of systematic review*
Defining eligibility criteria and screening studies	No – *basic general step of systematic review*
Data extraction	Yes – *new data extraction categories and items are needed*
Risk of bias assessment	Yes – *risk of bias tools need to be adapted to prevalence study designs*
Synthesis	Yes – *meta-analyses need to account for ceilings and floors for prevalence and levels of exposure*
Certainty of body of evidence assessment	Yes – *certainty assessment frameworks need to be adapted for prevalence evidence*

These adaptations amount to a novel, preliminary framework for conduct of systematic reviews of studies of prevalence of occupational exposures. We detail the areas in which new methods needed to be developed below.

#### Question formulation

Systematic reviews of environmental and occupational health evidence usually operationalise their research question in terms of a “PECO” statement, that characterises the population (P), exposure (E), comparator (C), and outcome (O) of interest in the review. However, the WHO/ILO systematic reviews of prevalence of exposures differ from reviews of effects of exposures, as they only capture the population and exposure; there is neither an outcome, nor a comparator of concern when prevalence of exposure alone is the issue of interest. Exposure prevalence can be measured in relation to an exposure limit, such as a limit of detection (i.e., the smallest amount of analyte which can be distinguished) or quantification (i.e., the mass of analyte equal to 10 times the standard error of the calibration graph divided by the slope), but this is different to a comparator. For example, the protocols for the WHO/ILO Joint Estimates systematic review of exposure, specified the types of populations: studies of working-age (≥15 years) workers in the formal and informal economy, with participants residing in any Member State of WHO and/or ILO, and working in any industrial setting or occupation. For the exposures, studies to be included were those that defined exposures in accordance with our pre-specified definitions (see [9] for more details about the systematic reviews).

#### Risk of bias assessment

Bias occurs when methods for generating or interpreting data in a study lead to systematic error in its findings. Assessing the risk of bias in individual studies is a critical part of systematic review, to account for the potential impact of the biases on the body of evidence. The need for risk of bias assessments was recognised in a recent systematic review on mercury biomarkers in human populations by Basu and colleagues [1], who adapted the US National Toxicology Program Office of Health Assessment and Translation (NTP OHAT) [7] tool to prevalence studies. While the nature of their adaptation is not fully documented, it seems designed to assess frequency of specific methodological approaches in prevalence studies rather than being targeted at assessing potential for systematic error in results. Two other tools for assessing prevalence studies, that are commonly cited, have been provided by Hoy et al. [2] and Munn et al. [6]. These tools also have important limitations in terms of assessing risk of bias, which we summarise in Table 2. Overall, there is a need to update critical appraisal tools for prevalence studies to reflect current understanding of best practice in risk of bias assessment in quantitative systematic reviews, adapted for the specific context of studies of prevalence of exposures.

**Table 2:** Previous risk of bias assessment tools for prevalence studies and their limitations

Tool	Description	Limitations in application to systematic reviews of prevalence studies
Hoy et al. 2012 [2]	A checklist intended to facilitate assessment of risk of bias in prevalence studies included in a systematic review, covering internal and external validity via 11 questions with yes/no answers.	• The tool appropriately focuses on internal validity. However, it is not clear how questions relate to risk of bias (e.g., “Were data collected directly from the subjects?”), and therefore whether the tool is sufficiently extensive in coverage of bias issues. • Only having yes/no answers may not be nuanced enough for capturing a range of risk of bias, especially for questions that may not have a clear yes/no answer (e.g., “Was the length of the shortest prevalence period for the parameter of interest appropriate?”). • It is also not clear how a range of yes/no answers aggregates into an overall risk of bias rating. • The tool is designed for studies of prevalence of disease rather than prevalence of exposures.
Munn et al. 2014 [6]	A questionnaire intended to facilitate critical appraisal of prevalence studies in a systematic review. Provides 10 questions with “yes”, “no”, “unclear”, or “not applicable” as answers.	• The tool focuses on a range of important characteristics of study quality; however, some of these characteristics do not relate to internal validity (e.g., “Was the sample size adequate?”). It is not therefore clear how the tool can provide an accurate account of vulnerability of studies to systematic error for the purposes of a systematic review. • The tool omits important bias issues such as selective reporting, and lacks transparency, as providing justification for judgements does not seem to be required. • The tool is designed for studies of prevalence of disease rather than prevalence of exposures.

Using existing tools as a template and building on current understanding of best practice in assessing internal validity of studies in systematic reviews, WHO and ILO, supported by individual experts, developed the RoB-SPEO (Risk of Bias in Studies estimating Prevalence of Exposure to Occupational risk factors) tool [10], as part of the WHO/ILO Joint Estimates, while introducing some new features specifically for the assessment of studies of the prevalence of exposures. RoB-SPEO adopts an approach to appraising studies that targets the internal validity of studies via a domain-based assessment of risk of bias [10]. This approach is more consistent with the state of science in evidence synthesis than the tick-box approaches adopted by some existing tools (Table 2). Additionally, the WHO/ILO systematic reviews separate assessment of risk of bias and assessment of certainty of evidence, which has also been done by some established systematic review frameworks, including those of the Cochrane Collaboration (https://www.cochrane.org/) and the Navigation Guide (https://prhe.ucsf.edu/navigation-guide), but not others. Similar to existing approaches, the WHO/ILO approach fully recognises that risk of bias assessments are based on individual, subjective judgments that need to be rendered transparent and operationalised into a reasonable working model describing the potential for systematic error in the results of a study. Furthermore, the WHO/ILO tool is specifically tailored to the study designs used for prevalence studies of occupational exposures. A recent performance assessment of the RoB-SPEO tool found that it places similar burden on assessors as other tools do, it achieved good inter-rater agreement, and user experience was generally positive; the assessment also identified next steps in tool development [5].

The assessment of external validity at the level of the individual study is a recognised need in systematic review [3]. In general, external validity is only currently assessed at the level of the body of evidence, with the Grading of Recommendations Assessment, Development, and Evaluation (GRADE) approach, the Navigation Guide, and the NTP OHAT handbook assessing external validity under the indirectness domain of the certainty assessment. More explicit procedures for assessing external validity for individual studies could be especially valuable for systematic reviews of prevalence studies, as it is one of the most important aspects of a prevalence study. However, given the lack of precedent at the time of developing the WHO/ILO prevalence systematic review protocols, this ended up falling outside the scope of the project, and we have no direct experience on which to draw to make specific recommendations in this regard. This should be addressed in future work.

#### Synthesis

Unlike for systematic reviews of effect of an exposure, measures of prevalence and levels of exposure are subject to ceiling and floor effects. It can never be concluded that less than 0% or more than 100% of a population are exposed to a risk factor; nor can a level of exposure be less than zero. Any framework for systematic review of studies of prevalence of exposure should guide reviewers to avoid drawing statistically incorrect conclusions. Statisticians can perform logit or double arcsine transformations in quantitative meta-analyses of prevalence estimates to prevent the pooled confidence intervals from exceeding the floor or ceiling, and to stabilize the variance. Confidence intervals for prevalence estimates can be skewed (sometimes highly so), and software to pool such estimates must therefore be able to accurately pool skewed data. Software commonly used for systematic reviews of effect, such as RevMan for Cochrane Reviews, often cannot be used to pool such skewed data; Meta-XL is one software that can be used. “Expected heterogeneity” is defined as the “real and non-spurious heterogeneity (i.e., variability) that can be expected in the prevalence of exposure, within or between individual persons, because exposure to the risk factor may change over space and/or time” ([8], p3). When “expected heterogeneity” is high, statistical heterogeneity can genuinely (non-erroneously) be very high in meta-analyses, so high statistical heterogeneity is not necessarily problematic and could be explored in relevant subgroup analyses (e.g., by occupation and industrial sector).

#### Rating the certainty of the body of evidence

Approaches developed specifically within the field of environmental and occupational health for assessing certainty of the body of evidence in studies of the effect of environmental and occupational exposures on health outcomes cannot directly be applied to systematic review of prevalence studies of environmental and occupational exposures. A recent review of certainty of evidence assessment tools for systematic reviews of environmental health risk assessment identified tools that conduct or provide methodological input for performing environmental health hazard assessments [11], including the Navigation Guide certainty of evidence tool (https://prhe.ucsf.edu/navigation-guide), and the GRADE Working Group tool for assessing certainty of evidence in environmental and occupational and occupational health (https://www.gradeworkinggroup.org/). However, these tools were developed to support certainty of evidence assessments for systematic reviews of the *effect* of environmental and occupational exposures on health outcomes (or their association); they may, therefore, require modification to be applicable to systematic review of prevalence studies of environmental and occupational exposures. To fill the gap, WHO, supported by individual experts, has developed QoE-SPEO (Quality of Evidence in Studies estimating Prevalence of Exposure to Occupational risk factors) [8], an approach for assessing certainty of evidence in studies estimating prevalence of exposure to occupational risk factors, including external validity (indirectness), inconsistency, and publication bias. Relevant steps, domains and components from GRADE were adopted or adapted for QoE-SPEO.

Additionally, this new approach encompasses the novel concept of “expected heterogeneity”, plus a novel first step of the assessment to rate this heterogeneity. This is an important addition; it acknowledges that some prevalence measures will be expected to vary (e.g., between and across workplaces, work tasks and time), and certainty of evidence should not be considered poorer when heterogeneity is found where it is expected. QoE-SPEO has been applied in the two WHO/ILO Joint Estimates systematic reviews of prevalence published to date.

### Next steps: continuing the progression towards a full framework

The WHO/ILO Joint Estimates have identified the need for a dedicated systematic review framework (i.e., standard methods plus standard tools and approaches) to provide standards and guidance on how to conduct systematic reviews of prevalence studies of exposures in environmental and occupational health. Such a framework will be key in reducing methodological inconsistencies and improving the quality of systematic reviews of prevalence studies of exposures, and it will aid their rigorous, systematic and transparent conduct. Existing methods have been adapted and new tools and approaches innovated and developed as part of a framework used in the WHO/ILO Joint Estimates. As noted above, in addition to pilot tests [10], the performance of the RoB-SPEO tool was also comprehensively evaluated in terms of assessor burden, inter-rater agreement and user experience across four systematic reviews [5]. Additionally, the QoE-SPEO approach was pilot tested and case studies of user experience were also conducted across two systematic reviews [8]. These assessments and feedback have provided evidence supporting the use of these new tools/approaches, and they have also been applied across five systematic reviews for the WHO/ILO Joint Estimates (see [9]). However, further developments are needed, which we identify in Table 3. As an overall outcome, a handbook could be produced as a guide to carrying out systematic reviews of this nature. Reporting guidelines could also be developed as part of the framework. The applicability of the new framework for conducting rapid and scoping reviews could also be explored. A framework that enables researchers to conduct systematic reviews along a specified and ordered process can also improve efficiency in research by reducing resources needed (e.g., researchers’ time), thereby potentially enabling more researchers to conduct such systematic reviews, including those working in settings with restrained resources. The new framework will need to be flexible for different research questions and should develop with time, as more researchers test it. Concerns about overly-rigid, algorithmic, or box-checking approaches to conducting various aspects of a systematic review are merited (e.g. [12]); however, we would argue that sufficient structure needs to be in place to allow for methods to be transparent, comparable, and reasonably reproducible. An appropriate balance between flexibility and structure needs to be struck.

**Table 3:** Next steps by systematic review framework step

Framework step	Next steps (final products: handbook and reporting guidelines)
Specify research question	• Improvement of research question formulation
Protocol	• Template for protocols for systematic reviews of studies of prevalence of exposure • Publication of protocols in a peer-reviewed journal
Systematic search	• Development of standard search strings and specialized search filters • Identification and development of specific academic and grey literature databases to search • Guidance on including modelling versus empirical data • Automated updating of searches
Defining eligibility criteria and screening studies	• Guidance to help reviewers produce eligibility criteria that are sufficiently detailed on exposure measurements and methods
Data extraction	• Template for extracting data from studies of prevalence of exposure • Guidance to include information on specific types of bias for which to extract data (e.g., numerator-denominator bias)
Risk of bias assessment	• Testing and further development of RoB-SPEO (more details in [10])
Synthesis	• Guidance on what evidence should be pooled and what should not (quantitative meta-analysis versus narrative synthesis) • Guidance on how to pool skewed data, with ceiling and floor (0 and 100% exposed), including which software to use • Guidance on interpreting pooled prevalence estimates, including model heterogeneity and skewed pooled confidence intervals
Certainty of body of evidence assessment	• Testing and further development of QoE-SPEO (more details in [8])

The new framework for systematic reviews of environmental and occupational exposures should be consistent with established systematic review practices, as outlined in the Cochrane Handbook (https://training.cochrane.org/handbook), and adapted for the environmental and occupational health context by the WHO Chemical Risk Assessment Network [14], NTP OHAT [7], the Navigation Guide (https://prhe.ucsf.edu/navigation-guide), and the COSTER recommendations [13]. This is because systematic reviewers may want to conduct systematic reviews of studies of environmental and occupational exposure prevalence, and of studies on the effect of the environmental and occupational exposure on a health outcome, in a harmonized manner and in tandem (as done for the WHO/ILO Joint Estimates). Tools for assessing risk of bias and certainty of evidence of prevalence studies, in general and for environmental and occupational exposures, are currently missing from the toolkit of epidemiologists, prompting the development of RoB-SPEO and QoE-SPEO. These will provide a better understanding of quantitative evidence synthesis methods for exposure studies and more consistency across systematic reviews. Both tools have been applied in the WHO/ILO Joint Estimates; however the validity of both approaches needs further testing, and both will benefit from further development over time.

## Conclusions

Attention and efforts are needed from policy and scientific communities globally to develop standards and tools or approaches for conducting and reporting high-quality systematic reviews of prevalence studies of exposure to environmental and occupational risk factors, as well as undertaking to test and evaluate those that are being introduced. Standards, agreed across disciplines (e.g. Epidemiology, Exposure Science, and Toxicology), will help improve the global evidence and knowledge base. The same standards are likely to be applicable to systematic reviews of nutritional, behavioural and, perhaps, social risk factors to human health. Policy and scientific communities (exposure scientists in particular) hold key knowledge and expertise to further develop the preliminary standards produced by WHO and partners.

## Data Availability

Not applicable.

## References

[1] Basu N, Horvat M, Evers DC, Zastenskaya I, Weihe P, Tempowski J (2018). A state-of-the-science review of mercury biomarkers in human populations worldwide between 2000 and 2018. Environ Health Perspect.

[2] Hoy D, Brooks P, Woolf A, Blyth F, March L, Bain C (2012). Assessing risk of bias in prevalence studies: modification of an existing tool and evidence of interrater agreement. J Clin Epidemiol.

[3] Jung A, Balzer J, Braun T, Luedtke K (2022). Identification of tools used to assess the external validity of randomized controlled trials in reviews: a systematic review of measurement properties. BMC Med Res Methodol.

[4] Lioy PJ, Smith KR (2013). A discussion of exposure science in the 21st century: a vision and a strategy. Environ Health Perspect.

[5] Momen NC, Streicher KN, da Silva DTC, Descatha A, Frings-Dresen MH, Gagliardi D, et al. Assessor burden, inter-rater agreement and user experience of the RoB-SPEO tool for assessing risk of bias in studies estimating prevalence of exposure to occupational risk factors: an analysis from the WHO/ILO Joint Estimates of the Work-related Burden of Disease and Injury. Environ Int. 2022;158:107005. 10.1016/j.envint.2021.107005 PMC8685606 34991265

[6] Munn Z, Moola S, Riitano D, Lisy K (2014). The development of a critical appraisal tool for use in systematic reviews addressing questions of prevalence. Int J Health Policy Manag.

[7] Office of Health Assessment and Translation (2015). OHAT risk of Bias rating tool for human and animal studies.

[8] Pega F, Momen NC, Gagliardi D, Bero LA, Boccuni F, Chartres N (2022). Assessing the quality of evidence in studies estimating prevalence of exposure to occupational risk factors: the QoE-SPEO approach applied in the systematic reviews from the WHO/ILO joint estimates of the work-related burden of disease and injury. Environ Int.

[9] Pega F, Momen NC, Ujita Y, Driscoll T, Whaley P (2021). Systematic reviews and meta-analyses for the WHO/ILO joint estimates of the work-related burden of disease and injury. Environ Int.

[10] Pega F, Norris SL, Backes C, Bero LA, Descatha A, Gagliardi D (2020). RoB-SPEO: a tool for assessing risk of bias in studies estimating the prevalence of exposure to occupational risk factors from the WHO/ILO Joint Estimates of the Work-related Burden of Disease and Injury. Environ Int.

[11] Rooney AA, Cooper GS, Jahnke GD, Lam J, Morgan RL, Boyles AL (2016). How credible are the study results? Evaluating and applying internal validity tools to literature-based assessments of environmental health hazards. Environ Int.

[12] Steenland K, Schubauer-Berigan MK, Vermeulen R, Lunn RM, Straif K, Zahm S (2020). Risk of bias assessments and evidence syntheses for observational epidemiologic studies of environmental and occupational exposures: strengths and limitations. Environ Health Perspect.

[13] Whaley P, Aiassa E, Beausoleil C, Beronius A, Bilotta G, Boobis A (2020). Recommendations for the conduct of systematic reviews in toxicology and environmental health research (COSTER). Environ Int.

[14] World Health Organization (2021). Framework for the use of systematic review in chemical risk assessment.

